# Osteoblastic heparan sulfate glycosaminoglycans control bone remodeling by regulating Wnt signaling and the crosstalk between bone surface and marrow cells

**DOI:** 10.1038/cddis.2017.287

**Published:** 2017-06-29

**Authors:** Rafik Mansouri, Yohann Jouan, Eric Hay, Claudine Blin-Wakkach, Monique Frain, Agnès Ostertag, Carole Le Henaff, Caroline Marty, Valérie Geoffroy, Pierre J Marie, Martine Cohen-Solal, Dominique Modrowski

**Affiliations:** 1 Inserm UMR-1132, BIOSCAR, Paris, France; 2 Université Paris Diderot, Sorbonne Paris Cité, Paris, France; 3 CNRS, UMR 7370, LP2M, Faculté de médecine, 28 avenue de Valombrose, Nice, France; 4 Université Nice Sophia Antipolis, Parc Valrose, Nice, France; 5 CNRS, USR3695, Gif-sur-Yvette, France

## Abstract

Stimulating bone formation is an important challenge for bone anabolism in osteoporotic patients or to repair bone defects. The osteogenic properties of matrix glycosaminoglycans (GAGs) have been explored; however, the functions of GAGs at the surface of bone-forming cells are less documented. Syndecan-2 is a membrane heparan sulfate proteoglycan that is associated with osteoblastic differentiation. We used a transgenic mouse model with high syndecan-2 expression in osteoblasts to enrich the bone surface with cellular GAGs. Bone mass was increased in these transgenic mice. Syndecan-2 overexpression reduced the expression of receptor activator of NF-*k*B ligand (RANKL) in bone marrow cells and strongly inhibited bone resorption. Osteoblast activity was not modified in the transgenic mice, but bone formation was decreased in 4-month-old transgenic mice because of reduced osteoblast number. Increased proteoglycan expression at the bone surface resulted in decreased osteoblastic and osteoclastic precursors in bone marrow. Indeed, syndecan-2 overexpression increased apoptosis of mesenchymal precursors within the bone marrow. However, syndecan-2 specifically promoted the vasculature characterized by high expression of CD31 and Endomucin in 6-week-old transgenic mice, but this was reduced in 12-week-old transgenic mice. Finally, syndecan-2 functions as an inhibitor of Wnt-β-catenin–T-cell factor signaling pathway, activating glycogen synthase kinase 3 and then decreasing the Wnt-dependent production of Wnt ligands and R-spondin. In conclusion, our results show that GAG supply may improve osteogenesis, but also interfere with the crosstalk between the bone surface and marrow cells, altering the supporting function of osteoblasts.

Improving bone formation is an important issue to rescue bone loss in aging patients or to repair bone defects after fracture or tumor resection. Glycosaminoglycans (GAGs) are key component of the bone matrix and cell surface that modulate the bioavailability and activity of various osteoclastic and osteogenic factors. Synthetic sulfated GAGs showed osteogenic properties *in vitro* and were proposed to be useful for biomaterial coating; however, contrasting results were obtained and the effects of GAGs on bone formation and resorption are still unclear.^1, 2^ Moreover, GAG accumulation was shown to have a role in the bone diseases associated with mucoplysaccharidoses or Leri pleonosteosis.^3, 4^

In the various studies, only soluble or matrix-associated GAGs were considered. To design more optimal GAGs for clinical use in bone regeneration, we need to better understand the functions of endogenous cellular GAGs. Moreover, GAGs are unique to each cell type because they depend on the assembly machinery and modifying enzymes and the expression pattern of the proteoglycans. GAGs at the surface of osteoblastic cells could be major factors in the bone surface environment.

Recently, sulfated hyaluronan and chondroitin sulfate were reported to inhibit sclerostin and to enhance bone regeneration in diabetic rats.^5^ Thus, a key function of GAGs in fostering osteogenesis may involve modulating Wnt signaling. Indeed, in addition to sclerostin activity, that of Wnt proteins and many other Wnt modulators also depends on interactions with proteoglycans.^6^

Syndecans are cell-surface heparan sulfate proteoglycans (HSPGs). They are low-affinity co-receptors with roles in docking, protection and concentration of their ligands. Syndecans also interact with high-affinity receptors and integrins to modulate intracellular signaling.^7, 8^ Syndecans positively or negatively modulate Wnt signaling.^9, 10^ Among the four syndecans, syndecan-2 was especially linked to osteoblastic differentiation during mouse development and in adult bone. In the embryo, syndecan-2 is expressed in the periosteum at the onset of osteogenesis, and its expression increases during osteoblast differentiation.^11^ Syndecan-2 is upregulated by osteogenic factors such as bone morphogenic protein-2 and Runx2.^12, 13^ Hence, the syndecan-2 level appears to be tightly controlled in osteoblastic cells. Overexpression of syndecan-2 in osteosarcoma cells alters multiple pathways involving PI3K, mitogen-activated protein kinases, nuclear factor kappa-B (NF-*k*B) and protein kinase C *δ*, and also canonical and non-canonical Wnt pathways.^14, 15, 16^ On another hand, syndecan-2 supports neo-angiogenesis during development in zebrafish and in tumors, whereas shed syndecan-2 alters angiogenesis through the inhibition of endothelial cell migration.^17, 18, 19^ These data suggest that osteoblastic syndecan-2 could have a role in the relationship between angiogenesis and osteogenesis.^20, 21, 22^

Here, we used a transgenic mouse model with syndecan-2 expression increased specifically in osteoblasts to identify the role of this proteoglycan and the associated GAGs during bone remodeling. Syndecan-2 overexpression enriched the bone surface with heparan sulfate and resulted in increased bone mass because of a strong inhibition of resorption. Syndecan-2 overexpression enhanced apoptosis of bone marrow cells and decreased osteoblast and osteoclast precursor populations. Syndecan-2 upregulated the pro-osteogenic vasculature in young mice but did not prevent the loss of this specific endothelium with age. It downregulated the Wnt–β-catenin–T-cell factor (TCF) pathway, thereby changing the osteoblastic environment. Our data identify osteoblastic heparan sulfate GAGs as novel modulators of the crosstalk between osteoblasts and their microenvironment.

## Results

### Higher level of syndecan-2 and heparan sulfate GAGs at the bone surface increased bone mass

**Figure 1:**
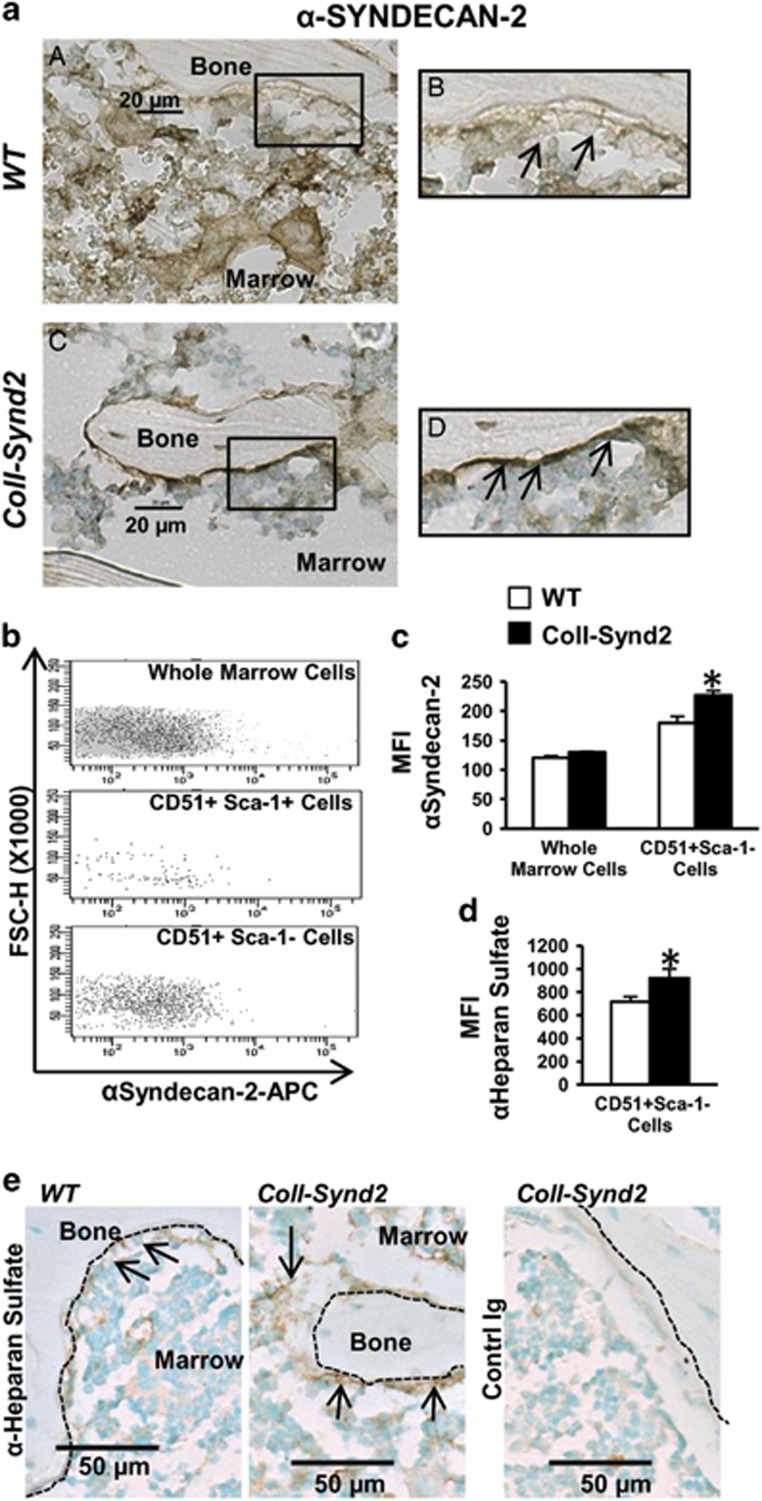
Syndecan-2 overexpression enriched bone surface with heparan sulfate glycosaminoglycans. (**a**) Immunohistological analysis of syndecan-2 in paraffin-embedded vertebra from 2-month-old WT (**a** and **b**) or ColI-Synd2 (**c** and **d**) mice. (**b**) Flow cytometry analysis of syndecan-2 expression. Marrow cells from 6-week-old mice were flushed from long bones and labeled with surface markers to select osteoblast precursors (CD51^+^Sca-1^+^ cells) or osteoblasts (CD51^+^Sca-1^−^ cells). Fluorescence intensity *versus* structural parameter of the cells (FSC-H) was plotted. (**c**) The median fluorescence intensity (MFI) of syndecan-2 labeling was recorded in different cell populations from WT or ColI-Synd2 mice. Data are mean±S.E.M. (*n*=7 WT and three ColI-Synd2 mice). (**d**) The MFI of heparan sulfate labeling was recorded in CD51^+^Sca-1^−^ cells from the marrow of WT or ColI-Synd2 mice. Data are mean±S.E.M. (*n*=4 WT and four ColI-Synd2 mice). (**e**) Immunohistological analysis of heparan sulfate chains in paraffin-embedded vertebra from 2-month-old WT or ColI-Synd2 mice. The photos presented are representative views of syndecan-2 or heparan sulfate staining. Arrows indicate syndecan-2- or heparan sulfate-expressing cells. Dashed lines indicate bone surfaces. *Indicates significant difference between WT and ColI-Synd2 (*P*<0.05)

**Figure 2:**
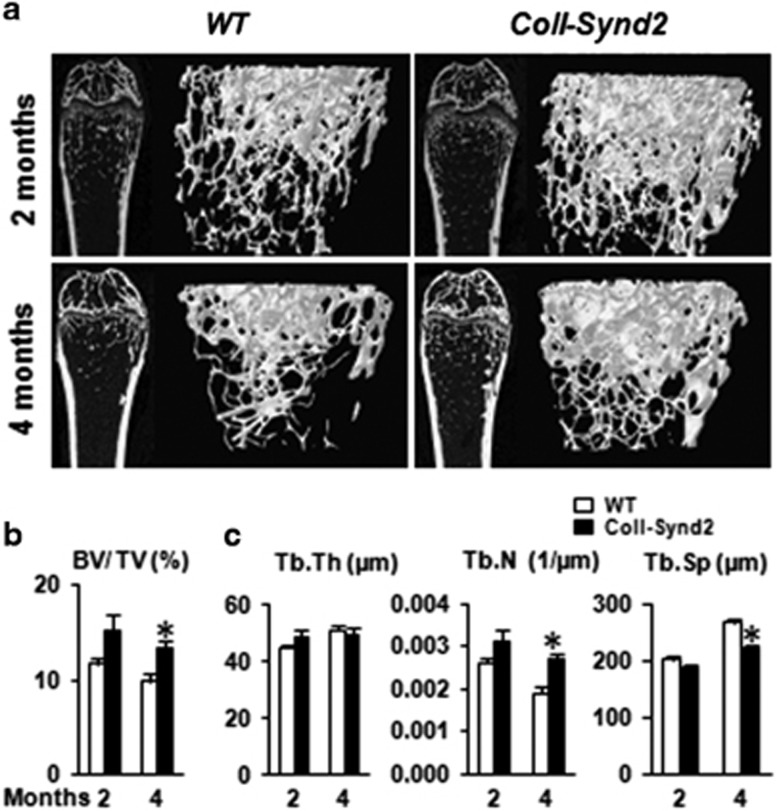
Syndecan-2 overexpression in osteoblasts resulted in higher bone mass. Micro-CT analysis of femurs. (**a**) Representative sections and 3D images of trabecular bone in femurs from male mice. Variations in trabecular bone volume corrected by tissue volume (BV/TV; **b**) and trabecular thickness (Tb.Th), separation (Tb.Sp) and number (Tb.N; **c**) in 2- and 4-month-old mice. Data are mean±S.E.M. (*n*=5 at 2 months; *n*=10 at 4 months). * indicates significant difference between WT and ColI-Synd2 (*P*<0.05)

Syndecan-2 was detected in bone marrow cells (BMCs), osteoblasts and osteocytes In wild-type (WT) mice (Figure 1a). We generated the C57BL/6-B6D2 Tg(ColI(2.3)-SCD2) (ColI-Synd2) transgenic mouse model by using the syndecan-2 sequence under control of the 2.3-kb fragment of collagen I promoter that drives transgene expression in osteoblasts.^23^ Transgenic mice showed increased number of cells with high level of syndecan-2 near the bone surface (Figure 1a). We collected BMCs from WT or ColI-Synd2 mice to further determine the levels of syndecan-2 by flow cytometry (Figures 1b and c). Only very few of the early osteoblast precursors with CD51 and Sca-1 at their surface (CD51^+^Sca-1^+^ cells) expressed syndecan-2 in WT as in ColI-Synd2 mice. The median fluorescence intensity of syndecan-2 labeling was not significantly modified when considering the whole BMC population from WT or ColI-Synd2 mice (Figure 1c). At the opposite, the osteoblasts (CD51^+^Sca-1^−^ cells) in the marrow of transgenic mice had a significant increase in syndecan-2 levels as compared to osteoblasts from WT mice (Figure 1c). Increased expression of syndecan-2 in CD51^+^Sca-1^−^ cells was associated with a increased level of heparan sulfate chains (Figure 1d). Indeed, heparan sulfate increased along the bone surface (Figure 1e). ColI-Synd2 mice from three strains from different founders developed normally without any significant differences in bone mineral density (Supplementary Figure 1). We used the transgenic strain with the highest syndecan-2 expression for micro-CT analyses to assess the effect of increased GAG level on bone mass in 2- (young) and 4-month-old (mature) mice (Supplementary Figure 1). Trabecular bone volume volume and number of trabeculae tended to increase in young transgenic males but were significantly higher in young females and in mature ColI-Synd2 male and females than in WT mice (Figures 2a–c and Supplementary Figure 2). Trabecula thickness was unchanged and trabecula separation was reduced in transgenic mice, especially at 4 months (Figure 2c). No modification of the cortical bone was observed in transgenic mice. Hence, increased level of the osteoblastic HSPG modified trabecular bone architecture.

### Syndecan-2 overexpression in osteoblasts inhibited bone resorption

**Figure 3:**
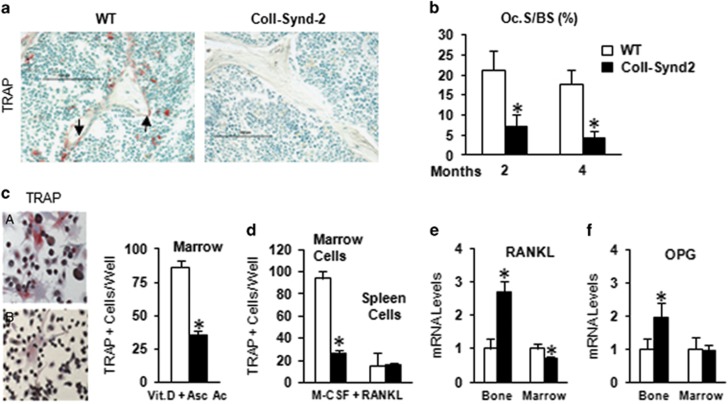
Syndecan-2 overexpression in osteoblasts inhibited bone resorption. (**a**) Detection of osteoclasts positive for TRAP (red cells) in femur sections from 10-week-old WT or ColI-Synd2 mice. (**b**) Quantification of proportion of bone surface with active osteoclasts (Oc.S/BS). Data are mean±S.E.M. (*n*=5 mice in each group). * indicates significant difference compared to controls (*P*<0.05). (**c** and **d**) Multinucleate TRAP-positive cells counted in bone marrow or spleen cells cultured with Vitamin D and ascorbic acid (**c**) or recombinant macrophage-colony-stimulating factor and RANKL (**d**) as indicated. Data are mean±S.E.M. from four different cultures. * indicates significant difference compared to controls (*P*<0.05). (**e** and **f**) RT-qPCR analysis of mRNA expression of RANKL and OPG in bone marrow cells from long bones or marrow-free bone tissue. Data are mean±S.E.M. (*n*=8 mice in each group). * indicates significant difference between WT and ColI-Synd2 (*P*<0.05). Arrows indicate TRAP positive osteoclasts

In adults, bone quantity results from the remodeling process, the first step of which is bone resorption by osteoclasts. ColI-Synd2 mouse bone showed a striking disappearance of osteoclasts with tartrate-resistant acid phosphatase (TRAP) activity (Figure 3a). The proportion of osteoclastic surface (Oc.S/BS) was significantly reduced in 2- and 4-month-old mice (Figure 3b). *Ex vivo* analyses revealed reduced formation of TRAP^+^ osteoclasts in cultures of BMCs from ColI-Synd2 long bones in the presence of vitamin D and ascorbic acid to promote the expansion of a supporting fibroblast-like layer (Figure 3c). The addition of pro-differentiating cytokines' macrophage-colony-stimulating factor and receptor activator of NF-*k*B ligand (RANKL) induced the formation of TRAP^+^ osteoclasts in cultures of spleen cells from WT and ColI-Synd2 mice, but did not rescue TRAP^+^ osteoclast formation in BMC cultures from ColI-Synd2 mice (Figure 3d), which indicates reduced number of osteoclast precursors in bone marrow of ColI-Synd2 mice. RANKL and its antagonist osteoprotegerin (OPG) are key factors controlling osteoclastogenesis. They are produced, in part, by osteoblasts and stromal cells within bone marrow. The RANKL mRNA level was lower in BMCs from ColI-Synd2 than in WT mice (Figure 3e), whereas in bone extracts containing mature osteoblasts and osteocytes, the RANKL level was increased in parallel with OPG level (Figures 3e and f). Hence, osteoblastic heparan sulfate may control osteoclastogenesis and bone remodeling.

### Syndecan-2 in osteoblasts modulated bone formation

**Figure 4:**
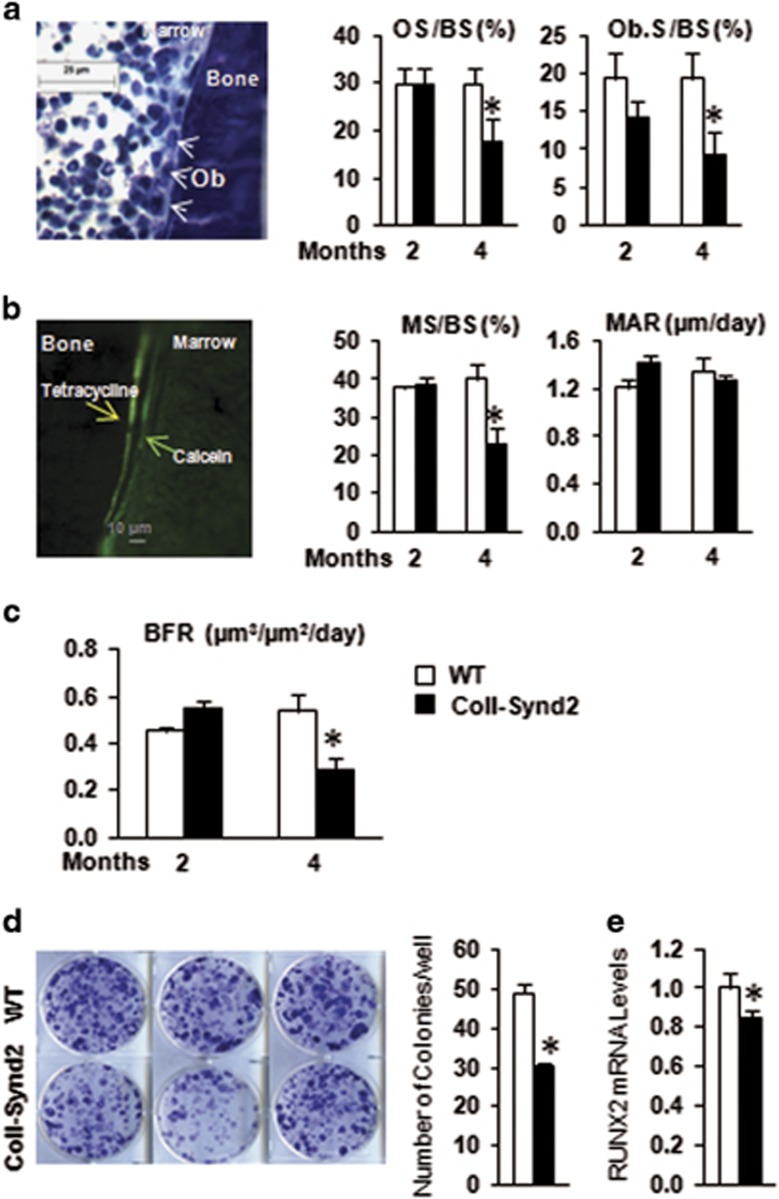
Syndecan-2 overexpression altered bone formation depending on mouse age. (**a**) Histomorphometric analysis of trabecular bone of male femurs in 2- and 4-month-old mice. Proportion of osteoid surface (OS/BS) and surface of active osteoblasts corrected by bone surface (Ob.S/BS) in femurs stained with toluidine blue. (**b**) Dynamic histomorphometric measurements of bone formation after calcein and tetracycline staining. (**c**) The extent of double labels and distance between the two stainings allowed for calculating the extent of mineralized surfaces (MS/BS), mineral apposition rate (MAR) and bone formation rate (BFR). Data are mean±S.E.M. (*n*=5 mice). (**d**) BMCs from WT or ColI-Synd2 mice were cultured for 11 days and stained for alkaline phosphatase activity for colony-formation unit (CFU)^−^ALP^+^ assay. Data are mean±S.E.M. number of colonies per well (*n*=5 independent cultures from five mice). (**e**) RT-qPCR analysis of RUNX2 mRNA expression in BMCS from long bones. Data are mean±S.E.M. (*n*=5 mice). * indicates significant difference between WT and ColI-Synd2 (*P*<0.05)

Syndecan-2 was found to be involved in signaling that favors osteoblastic activity.^24^ Here, we studied formation parameters using toluidine blue staining of osteoid tissue and osteoblasts (Figure 4a) and tetracycline and calcein injections to visualize the dynamic mineralization process (Figures 4b and c). The extent of bone surface with new matrix deposit, osteoid surface, number of active osteoblasts and mineralized surfaces was not significantly modified in 2-month-old ColI-Synd2 mice but were strongly reduced in 4-month-old ColI-Synd2 mice as compared to WT mice (Figures 4a and b). The mineral apposition rate was not significantly modified (Figure 4b). As a result, the global bone formation rate was decreased in 4-month-old ColI-Synd2 mice (Figure 4c). Hence, osteoblastic GAGs did not alter osteoblastic activity but downregulated osteoblast number. This latest effect did not appear to be related to altered proliferation or differentiation capacity of osteoblastic precursors as shown by BrdU incorporation and *in vitro* mineralization assay in mesenchymal cells from ColI-Synd2 or WT bone marrow (Supplementary Figure 3). However, the formation of ALP^+^ colonies (CFU^−^ALP^+^) was significantly impaired in cultures of BMCs from ColI-Synd2 mice as compared with WT mice (Figure 4d). In addition, the expression of the osteoprogenitor marker RUNX2 was significantly decreased in bone marrow extracts from transgenic mice (Figure 4e). Thus, GAGs at the bone surface increased osteoblast activity in younger mice, reduced osteoblast number in older mice and altered the pool of osteoblast precursors in bone marrow.

### Osteoblastic syndecan-2 promoted stromal-cell apoptosis

**Figure 5:**
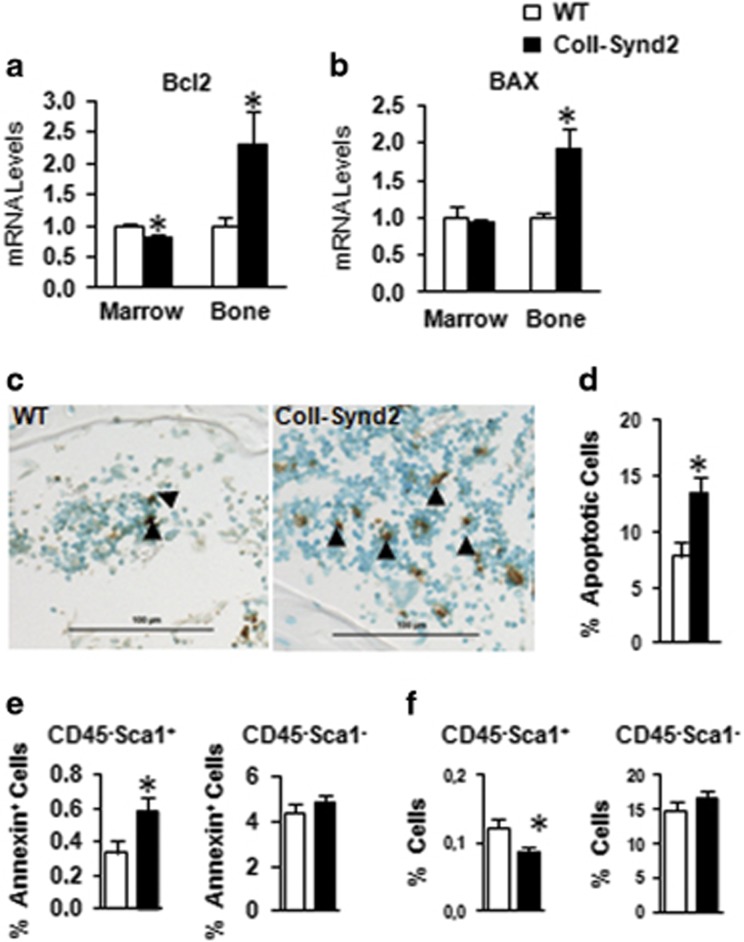
Osteoblastic syndecan-2 affected osteogenic precursor apoptosis. RT-qPCR analysis of mRNA expression of Bcl2 (**a**) and BAX (**b**) in BMCs or bone extracts from 4-month-old mice. Data are mean±S.E.M. (*n*=5 mice in each group). (**c** and **d**) TUNEL analysis of apoptosis in vertebra sections of 4-month-old mice. The results are the mean±S.E.M. of the % of TUNEL-positive BMCs in vertebra sections from three mice. (**e** and **f**) Quantified flow cytometry of apoptotic Annexin^+^ (**e**) and BMC populations (**f**) of stromal precursors (CD45^−^Sca-1^+^) and stromal mature cells (CD45^−^Sca-1^−^). Data are mean±S.D. percentage (*n*=5 mice). (**f**) Quantification of percentage of stromal precursors (CD45^−^Sca-1^+^) and mature stromal cells (CD45^−^Sca-1^−^). * indicates significant difference compared to controls (*P*<0.05). Arrowheads indicate apoptotic cells

Survival of osteoblasts and their precursors may affect global osteogenic activity. Syndecan-2 overexpression decreased cell survival and increased effector caspase activity of proliferating stromal C3H10½ cells but not confluent cells (Supplementary Figure 4); therefore, syndecan-2 overexpression may be associated with increased apoptosis. Consistently, the mRNA level of anti-apoptotic Bcl2 was decreased in bone marrow extracts and increased in bone cells of ColI-Synd2 mice; the level of the pro-apoptotic Bax was unchanged in BMCs and increased in bone cells (Figures 5a and b). TUNEL assay showed a significant increase in the rate of apoptotic cells within the bone marrow of ColI-Synd2 vertebra (Figures 5c and d). However, the number of osteocytes and rate of apoptotic osteocytes did not differ between control and transgenic mice (Supplementary Figure 5). These results support syndecan-2 not affecting the survival of mature osteoblasts but increasing the apoptosis of BMC populations. Accordingly, Annexin V binding was increased only in CD45^−^Sca-1^+^ mesenchymal progenitor populations but not in CD45^−^Sca-1^−^ mature cells isolated from ColI-Synd2 bone marrow (Figure 5e). Increased apoptosis was associated with a decreased rate of mesenchymal progenitors (CD45^−^Sca-1^+^) in the bone marrow of ColI-Synd2 mice (Figure 5f). Syndecan-2 may prevent osteoblasts from supporting the survival of mesenchymal stem/precursor cells in bone marrow.

### Osteoblastic syndecan-2 had an impact on pro-osteogenic vasculature

**Figure 6:**
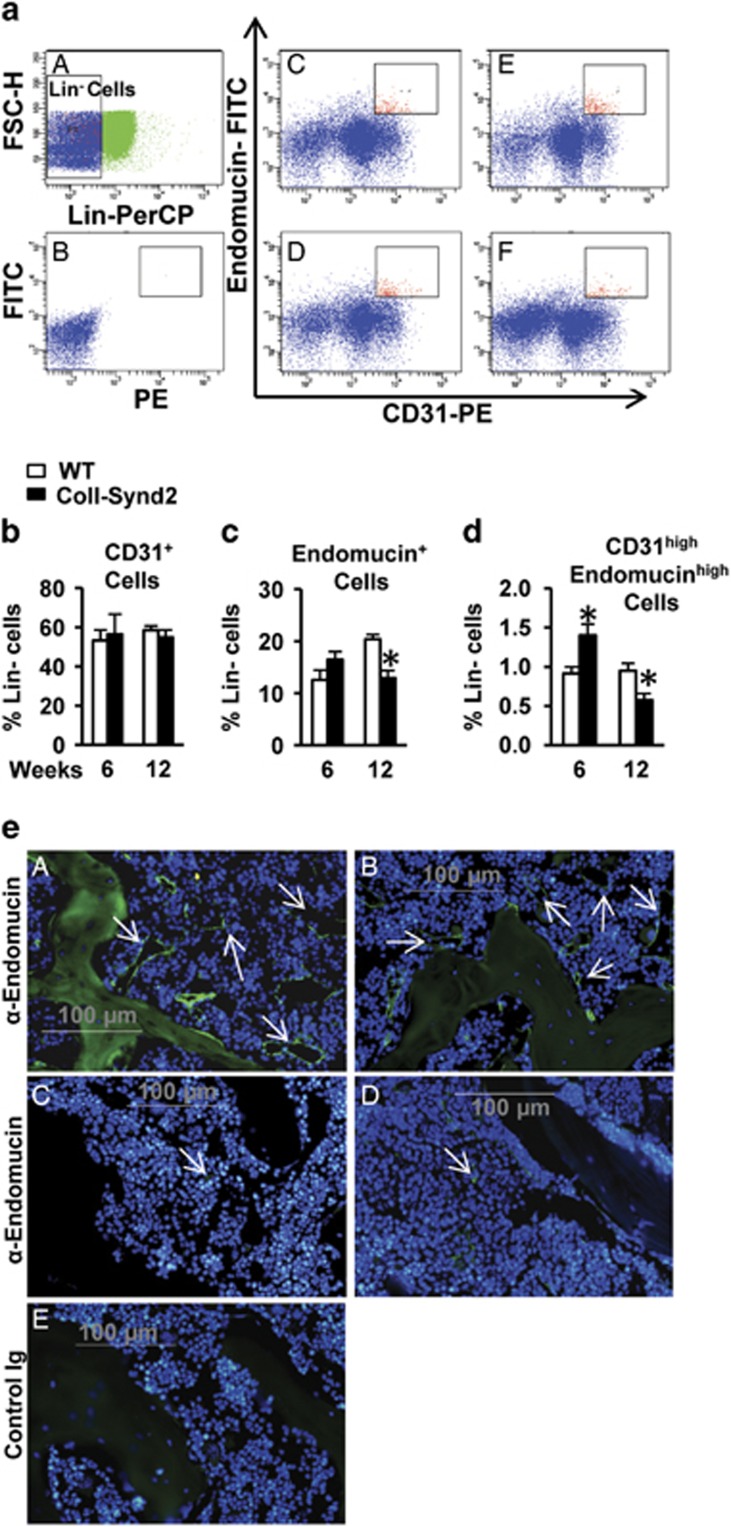
Syndecan-2 overexpression altered angiogenesis. Flow cytometric analysis of CD31 and Endomucin-stained marrow cells. (**a**) Cells that did not express the markers of hematopoietic lineages (blue gate, Lin^−^) have been selected for the analysis of CD31 and Endomucin expression. (**B** and **F**) Representative dot plots of CD31 and Endomucin staining in WT (**C** and **D**) or transgenic mice (**E** and **F**) at 6 weeks (**C** and **E**) or 12 weeks (**D** and **F**) of age. P5 gate was placed arbitrarily to select cells with the higher levels of CD31 and Endomucin. (**b**) Quantification of the % of Lin^−^ cells with CD31 staining. (**c**) Quantification of the % of Lin^−^ cells with Endomucin staining. (**d**) Quantification of the % of Lin^−^ cells that expressed high levels of CD31 and Endomucin (P5 gate). Results are the mean %±S.E.M. (seven WT and three ColI-Synd2 for 6-week-old mice; four WT and four ColI-Synd2 for 12-week-old mice). * indicates significant difference between WT and ColI-Synd2 (*P*<0.05). (**e**) Representative photos of analysis of Endomucin-expressing vessels within the bone marrow of 6-week-old (**A** and **B**) or 4-month-old (**C** and **D**) mice. Sections of paraffin-embedded vertebra from WT (**A** and **C**) or transgenic (**B** and **D**) mice were stained with an anti-endomucin antibody and green fluorescence-conjugated secondary antibody. DAPI was used to stain the nuclei. Control sections were incubated with rat Ig (**E**). Arrows indicate the vessels with Endomucin^+^ cells

Increased apoptosis of BMCs was observed as soon as at 2 months in transgenic mice and did not alone explain how syndecan-2 overexpression differently influenced the bone formation at 2 and 4 months. To further address this question, we examined whether syndecan-2 overexpression did modify the endothelium within the bone marrow using CD31 and Endomucin markers to study a pro-osteogenic endothelium that was previously shown to be characterized by high expression of CD31 and Endomucin (CD31^high^Endomucin^high^ cells; Figure 6a).^22^ We selected marrow cell populations that did not express lineage markers (Lin^−^ cells). The proportion of Lin^−^ cells that expressed CD31 was similar in WT and ColI-Synd2 mice (Figure 6b). At the opposite, the proportion of Lin^−^ cells that expressed Endomucin was significantly decreased in the marrow of 12-week-old transgenic mice as compared to WT mice at the same age (Figure 6c). The proportion of CD31^high^Endomucin^high^ cells was increased in 6-week-old but decreased in 12-week-old ColI-Synd2 mice (Figure 6d). Analyses by immunofluorescence illustrated that syndecan-2 overexpression did not prevent the loss of Endomucin^+^ endothelium within the marrow of 4-month-old mice (Figure 6e).

### Syndecan-2 overexpression in osteoblasts decreased Wnt/β-catenin signaling

**Figure 7:**
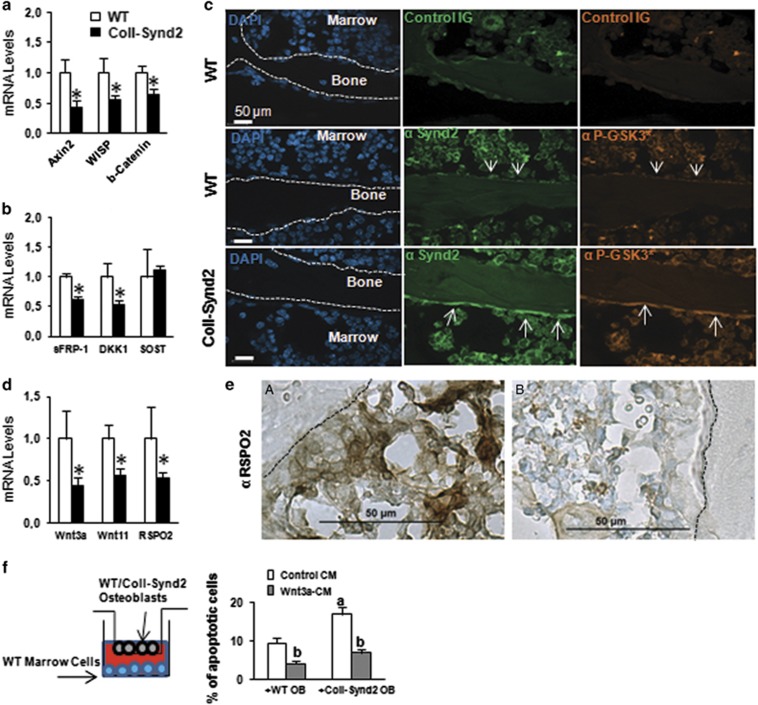
Syndecan-2 overexpression decreased Wnt/β-catenin signaling in osteoblasts. RT-qPCR analysis of Wnt target genes (**a**) and Wnt inhibitors (**b**) in bone extracts. Data are mean±S.E.M. (*n*=6 mice). * Indicates significant difference from controls (*P*<0.05). Immunofluorescence analysis of syndecan-2 expression and GSK3 activation in vertebra with the antibodies anti-syndecan-2 (*α*-synd2) and anti-phospho-GSK3 (Tyr279/Tyr216; *α*-PGSK3*). Cell nuclei were stained with DAPI. (**d**) RT-qPCR analysis of Wnt ligands and RSPO-2. (**e**) Expression of RSPO-2 in vertebra sections. Dashed lines indicate bone surface. (**f**) BMCs from WT mice were cultured with porous inserts containing WT or ColI-Synd2 osteoblasts with and without 15% Wnt3a-containing medium (Wnt3a-CM). Apoptotic BMCs were stained with the Sytox probe and counted. Data are mean±S.E.M. of the % of apoptotic cells (*n*=6 WT and 6 ColI-Synd2 OB cultures). ^a^ indicates a significant difference between culture with WT and ColI-Synd2 osteoblasts. ^b^ indicates a significant difference between Wnt3a-treated and non-treated cells. SOST, sclerostin

We next investigated whether the functions of osteoblastic GAGs were related to the modulation of Wnt activity. Basal and Wnt3a-induced activity of the TOPFlash reporter plasmid was reduced in syndecan-2-overexpressing C3H10½ cells (Supplementary Figure 6). This inhibitory effect was dependent on syndecan-2-associated GAGs since overexpression of a mutated syndecan-2, in which the three GAG-biding serines were replaced by alanines, did not induce the same modification of the TOPFlash activity (Supplementary Figure 6). Moreover, inhibition of sulfation of GAG chains with sodium chlorate increased basal and Wnt3a-dependent TOPFlash activity (Supplementary Figure 6). Therefore, the osteoblastic syndecan-2 appears to function as a Wnt signaling inhibitor. Consistently, the expression of the Wnt target genes *Axin2*, *WISP* and *β-catenin* was lower in bone extracts from ColI-Synd2 than in WT mice (Figure 7a). Syndecan-2-dependent changes in the expression of specific Wnt inhibitors, sFRP-1, DKK1 and sclerostin, could not explain this altered Wnt signaling in ColI-Synd2 mice (Figure 7b). Syndecan-2 may alter Wnt signaling by intracellular routes, as was previously shown.^15, 16^ Using an anti-phospho-GSK3 (Tyr279/Tyr216) antibody to label the active form of the kinase, serum-starved C3H10½ control cells showed inactivated GSK3 by the addition of serum; in contrast, GSK3 was activated in syndecan-2-overexpressing C3H10½ cells (Supplementary Figure 7). Syndecan-2 overexpression was also associated with enhanced phospho-GSK3 staining in osteoblasts along the bone surface of transgenic mice (Figure 7c). As a possible result of inhibition of the Wnt/β-catenin pathway, the expression of Wnt-dependent genes such as *Wnt3a*, *Wnt11* and *RSPO-2* were reduced in bone extracts of transgenic mice (Figure 7d). Decreased level of RSPO-2 in the osteoblastic microenvironment in ColI-Synd2 vertebra was confirmed by immunohistochemistry (Figure 7e). Increased level of GAG at the cell surface could also enhance the trapping capacities of the osteoblasts. Indeed, pre-incubation with C3H10½ cells that overexpressed syndecan-2 significantly reduced the ability of Wnt3a-containing medium to induce axin expression (Supplementary Figure 7). This effect was abolished when Wnt3a-containing medium was pre-incubated on C3H10½ cells that overexpressed the mutated syndecan-2 lacking heparan sulfate modifications (Supplementary Figure 7). To determine whether the modification of Wnt effectors around transgenic osteoblasts may contribute to the altered BMCs, we co-cultured WT or ColI-Synd2 osteoblasts in porous inserts with mesenchymal cells from WT bone marrow. BMC apoptosis was greater with syndecan-2-overexpressing osteoblasts than WT osteoblasts; the pro-apoptotic effect was rescued when the medium was supplemented with recombinant Wnt3a-containing medium (Figure 6f).

## Discussion

Synthetic GAGs have been considered promising compounds to improve biomaterial functions in the bone. To further define the usefulness of GAGs as an anabolic support, we investigated the function of endogenous cellular GAGs during bone remodeling by using a transgenic mouse model with increased syndecan-2 expression in osteoblasts. High syndecan-2 expression in osteoblasts enriched the bone surface with heparan sulfate and resulted in increased bone mass. Syndecan-2 overexpression inhibited resorption and decreased bone remodeling. The proteoglycan had a different impact on angiogenesis in young and mature mice. Syndecan-2 inhibited Wnt–β-catenin–T-cell factor pathway.

We used a ColI-Synd2 mouse model in which osteoblastic GAGs were specifically altered because sulfated GAG motives depend on specific cell machineries and determine the affinity for ligands and the distinct biological functions.^25, 26, 27^ The level of heparan sulfate was found enhanced at the bone surface in ColI-Synd2 mice. Hence, our mouse model provides insight into the specific role of this type of GAG in an osteoblastic environment.

Syndecan-2 overexpression resulted in higher bone mass in 2-month-old females and mature (4-month-old) adult mice. This finding is consistent with the sclerotic phenotype observed in Leri plenosteosis shown to be related to syndecan-2 gene duplication.^3^ In ColI-Synd2 mice, increased bone mass was associated with strong inhibition of the resorption. This indirect effect might be related to the reduced RANKL production in BMCs, with no effect on the balance of RANKL/OPG in osteoblast/osteocyte cells. We cannot exclude that heparan sulfate at the bone surface could positively or negatively affect osteoclast differentiation or activity as was previously proposed.^1, 28, 29, 30, 31^ Indeed, excess GAG in transgenic mice depleted the number of osteoclast precursors within the bone marrow.

Some evidence shows that, in addition to other marrow cell types and mature osteoblasts, stromal cells and immature osteoblasts produce chemokines that target circulating osteoclast precursors to increase their bone marrow migration, differentiation and survival.^32^ The decreased number of CFU^−^ALP^+^ cells we found in cultures of BMCs from transgenic mice as well as reduced number of Sca-1^+^CD45^−^ mesenchymal precursors may indicate that GAGs at the osteoblast surface modulate bone resorption by controlling BMC populations that support osteoclastogenesis.

Syndecan-2 overexpression affected bone formation depending on the age of mice. It did not alter osteoblast activity but reduced osteoblast number. Syndecan-2 has been described as a promoter of osteoblast adhesion to the matrix and a modulator of matrix deposition.^24, 33^ Our results are consistent with reports showing that heparan sulfate has an anabolic effect during bone regeneration in rat models of long bone or cranial defects.^34, 35^ During fracture repair, the ossification occurs through a callus formation and resembles the endochondral ossification process. In contrast, in mature animals, bone formation is part of the remodeling process and depends on coupling signals between osteoclasts and osteoblasts.^36^ The decreased bone formation rate in our 4-month-old transgenic mice probably resulted from a missing osteoclastic signal that impaired recruitment of osteoblast precursors. Increased apoptosis of mesenchymal precursors may also account for the reduced number of osteoblasts in older transgenic animals. Indeed, the heparan sulfate GAGs located at the interface between osteoblasts and BMCs seem to prevent osteoblasts, supporting the survival of BMC populations. Osteoblastic GAGs appear to affect specific BMC populations because the level of the apoptosis marker, Annexin V, was found to be associated with Sca-1^+^CD45^−^ but not more mature CD45^−^Sca-1^−^ BMCs from ColI-Synd2 mice. Syndecan-2 overexpression did not affect apoptosis of other cell populations such as CD11b^+^ or CD19^+^ B cells (data not shown). In another hand, syndecan-2 overexpression modified bone marrow vasculature. These results are consistent with previous data showing that syndecan-2 is involved in neo-angiogenesis during development.^18^ Here we show that syndecan-2 specifically promoted the CD31^high^Endomucin^high^ endothelium in youngest mice. This type of vessels mediates growth of the bone vasculature and supports osteogenesis.^22^ Hence, preserved bone formation in 2-month-old transgenic mice, despite osteoprogenitors' apoptosis, can be related to an increased pro-osteogenic endothelium. Our results showed that the proportion of CD31^high^Endomucin^high^ cells was increased in younger transgenic animals, whereas it was strongly decreased in mature transgenic mice. In another hand, the Endomucin^+^ bone vasculature decreased with age in WT mice, and this loss was not prevented in ColI-Synd2 mice. Hence, the decreased bone formation in 4-month-old ColI-Synd2 mice could be related to the reduction of the supportive action of CD31^high^Endomucin^high^ vasculature. Different mechanisms could be involved in this dual action of syndecan-2 on angiogenesis. Inhibition of bone remodeling in transgenic mice probably induced the maintenance of old osteoblasts with altered activity at the bone surface. Thus, syndecan-2 may be indirectly responsible for the modification of angiogenic factor production by osteoblasts. In another hand, shedding of syndecan-2 might be increased in older mice. This would contribute to switch the pro-angiogenic action of the proteoglycan into inhibition.^19^

Inhibition of Wnt signaling in osteoblasts contributed to the pro-apoptotic effect of syndecan-2 overexpression. Syndecan-2 expression modified intracellular signaling that affects the Wnt/β-catenin–T-cell factor pathway. In particular, syndecan-2 deregulated the GSK3 pathway, which may be related to the inhibitory effect of syndecan-2 on the PI3K level, as was previously reported in osteosarcoma cells.^15^ Wnt ligands secreted by osteoblasts have a crucial role in bone homeostasis.^37^ Therefore, inhibition of the expression of Wnt target genes, including Wnt ligands and RSPO, likely contributed to the altered microenvironment of osteogenic cells. However, Wnt and RSPOs are heparin-binding molecules. Our results suggest that high levels of proteoglycans may result in exaggerated capture of Wnt and other factors that are required to support Wnt signaling in BMCs. In the same way, sulfated hyaluronan coated on biomaterials was proposed to promote osteoblast function by binding sclerostin.^5^ Although the expression of Wnt inhibitors such as DKK1 and sFRP-1 was decreased in ColI-Synd2 mice, we cannot rule out that high levels of syndecan-2-conjugated heparan sulfate chains may promote sFRP-1 activity because heparin is responsible for sFRP-1 accumulation and stabilization.^38^ Moreover, RSPO is a DKK1 inhibitor.^39, 40^ Hence, decreased RSPO level could also favor DKK1 activity in the osteoblastic environment. Many genetic models have proven that Wnt signaling is required for stem-cell commitment, differentiation and osteoprogenitor survival. However, the physiological requirement for the extinction of Wnt signaling for the terminal maturation of osteoblasts was proposed from data showing that aberrant stabilization of β-catenin in precursors prevented the terminal step of osteoblastic maturation.^41, 42^ Overactivation of β-catenin in osteocytes and mature osteoblasts results in decreased bone quality and growth.^43^ Hence, inhibition of Wnt signaling can affect precursor cells within bone marrow and at the same time promote osteoblast activity.

In contrast with our results, heparan sulfate was shown to enhance the differentiation of osteoprogenitors and promote the proliferation of mesenchymal stem cells in culture systems.^44, 45^ Moreover, osteoporosis was found as a side effect of treatment with heparin that stimulated osteoclastogenesis by inhibiting OPG.^46, 47^ These discrepancies may be due to the different composition of the GAGs used in previous studies because the degree of sulfation affects the biological activity of these molecules.^27^ Osteoblastic GAGs should be better characterized at different ages and in particular pathological conditions to determine the optimal composition for GAG mimetics. Another way to interpret the different effects of GAG supply is that exogenous GAG could interfere or compete with the endogenous ones. Whether GAGs associated with or solubilized from a biomaterial could alter normal bone remodeling remains unknown.

## Conclusions

The ColI-Synd2 mouse model provided new insights into the specific activities of syndecan-2-conjugated heparan sulfate GAGs during bone remodeling. These GAGs at the surface of osteoblasts were key constituents in the osteoblast environment and controlled osteogenesis, osteoclastogenesis and bone remodeling in our model. Especially, syndecan-2 controlled the ability of osteoblasts to support other BMC populations such as stromal cells and mesenchymal precursors. The proteoglycan also modulated angiogenesis, altering a specific endothelium with pro-osteogenic properties. Osteoblastic GAGs were involved in regulating Wnt signaling and controlling Wnt effectors' production. Beyond promoting osteogenic properties, GAGs in biomaterials should provide an environment that can support stromal and hematopoietic cells of bone marrow.

## Materials and methods

### Animals

The entire protocol was performed in accordance with French Government Animal Welfare Policy and European Directive 86/609/EEC and was approved by the Ethics, Animal Care and Experimentation Committee of the Institute of Health and Medical Research at Lariboisière-Villemin (Paris, France). C57BL/6-B6D2 Tg(ColI(2.3)-SCD2) (ColI-Synd2) mice were generated as previously described by using the construct with N-cadherin cDNA replaced by human syndecan-2 cDNA.^48^ The mouse genotypes were determined by PCR amplification of tail DNA with 5′-TTGTATCCTCTTCGGCTGG-3′ and 5′-AGGCAGTTCTGATTGGCTGGG-3′ sequences as primers. Animals were not randomized. WT are littermate controls.

### Histomorphological analyses of the bone

Micro-CT analyses involved use of a SKYSCAN 1272 scanner (Bruker, Coventry, UK). Bones were placed vertically and scanned with the settings: aluminum filter, 0.5 mm; resolution, 6 *μ*m; energy, 70–90 kV; intensity, 100 *μ*A; and integration time, 170 ms. Reconstruction of femurs and analysis of bone volume involved the use of CTanalyser. 3D illustrations were created with DataViewer and 3D-Visualization software (CTvol). The histomorphometric variables were recorded in compliance with the recommendation of the American Society for Bone and Mineral Research Histomorphometry Nomenclature Committee.^49^ Given this recommendation and the type of mouse strain, five mice were included in each group for *μ*CT analyses at 2 months and 10 animals/groups at 4 months. The bone formation and resorption variables were measured in sections of methyl methacrylate-embedded femurs stained with toluidine blue to show osteoid tissues and osteoblasts or with naphthol 3-hydroxy-2-naphthoic acid 4-chloro-2-methylanilide (ASTR) phosphate for detecting mature osteoclasts with TRAP activity. Five animals/group were analyzed by two different investigators. One was blinded to the group allocation during the analysis. To evaluate bone formation rate, mice were intraperitoneally injected with double fluorescent labeling of tetracycline (20 mg/kg) and calcein (10 mg/kg; Sigma, St. Louis, MO, USA) at 5 days and 1 day before being killed. Measurements involved use of a polarizing microscope (Nikon, Tokyo, Japan) with a Retiga 2000 R (Q Imaging, Surrey, BC, Canada) and a software package developed for bone histomorphometry (Microvision, Lisses, France).

### Immunohistochemical analysis

Vertebrae from WT and ColI-Synd2 mice were fixed in 4% paraformaldehyde, decalcified and embedded in paraffin. Sections of 5 *μ*m were treated with 10 mM citrate buffer, pH 6, at 70 °C, saturated with goat serum and bovine serum albumin, incubated with the antibodies for control immunoglobulin, anti-syndecan-2, (366200, Invitrogen, Thermofisher, Montigny le Bretonneux, France), anti-R-spondin-2 (C-12; Santa Cruz Biotechnology, Dallas, TX, USA) or anti-heparan sulfate (F58-10E4; Amsbio, Cambridge, MA, USA), and then with horseradish peroxidase-conjugated secondary antibodies. Sections were counterstained with methyl green and observed under a polarizing microscope (Nikon) with a Retiga 2000 R camera (Q Imaging). For immunofluorescence of syndecan-2, activated glycogen synthase kinase 3 (GSK3) or Endomucin, sections were incubated with the antibody anti-syndecan-2 and anti-phospho-GSK3 (Tyr279/Tyr216; 5G-2F; Millipore, Guyancourt, France), or with anti-endomucin (V.7C7) then with DyLight488- and DyLight650-conjugated anti-immunoglobulin antibodies (Pierce, Thermofisher, Waltham, MA, USA). Nuclei were stained with DAPI. Sections were observed under a fluorescent microscope with the Apotome 1 system (Zeiss, Oberkochen, Germany) interfaced with Axiovision software and a × 20 objective.

### TUNEL assay

Paraffin-embedded vertebrae sections from 4-month-old mice were used for TUNEL assay (ApopTag Plus Peroxidase four to eight fields in sections by using the semi-automated software Bonolab (MicroVision). Results are presented as ratio of apoptotic BMCs to total cells or ratio of total or apoptotic osteocytes to the measured bone surface.

### Flow cytometry

BMCs were flushed from long bones, and red blood cells were lysed. All staining steps were performed at 4 °C in phosphate-buffered saline containing 1% fetal calf serum (FCS) and 2 mM EDTA with the antibodies anti-CD45 (30F11) and anti-Sca-1 (D7; BD Biosciences, Plymouth, UK). Cells were first labeled with cell-surface antibodies, and then with Annexin V according to the manufacturer’s procedure (BD Biosciences). To analyze syndecan-2 expression in osteoblastic cells we used anti-CD51 (RMV-7, Biolegend, San Diego, CA, USA) as osteoblast marker,^50^ anti-Sca-1 (D7, Miltenyi Biotec, Bergisch Gladbach, Germany) as precursor marker^51^ and anti-syndecan-2 (305515, R&D Systems, Minneapolis, MN, USA). To label endothelial cells we used anti-CD31 (390, BD Biosciences) and anti-endomucin (eBioV.7C7, eBioscience). To exclude mature hematopoietic lineages from the analysis, the marrow cells were labeled with a Lineage Cell Detection Cocktail-Biotin that contains biotin-conjugated monoclonal antibodies against the following: CD5, CD11b, CD45R, Anti-7-4, Anti-Gr-1 (Ly6G/C) and Anti-Terr-119 (Miltenyi Biotec). Cells were analyzed by using FACS Canto II, and the BD FACSDIVA software (BD Biosciences).

### RNA preparation from bone tissues

For each animal, BMCs were collected by centrifugation of long bones and suspended in 1 ml Trizol reagent (Invitrogen). Bone marrow-free bones were cut into pieces, immediately frozen in dry ice and ground with Trizol.

### Cell cultures

BMCs were flushed from long bones and cultured as described.^52^ Osteoblasts were obtained by migration from trabecular fragments of long trabecular bone.^53^ Murine pluripotent mesenchymal C3H10T1/2 cells were obtained from the ATCC, and then infected with the control or syndecan-2-coding lentiviral vector as described.^54^ Cells were cultured in Dulbecco’s modified Eagle’s medium (DMEM) supplemented with 10% FCS and antibiotics (100 IU/ml penicillin and 100 *μ*g/ml streptomycin; DMEM/FCS/P/S).

### *Ex vivo* osteoclastogenesis assay

Spleen or BMCs were isolated from 10-week-old WT and ColI-Synd2 male mice. Cell suspensions were obtained by using a 40-*μ*m nylon mesh cell strainer. After red blood cell lysis, cells were cultured in complete *α*-MEM medium with 10^−8^ M dihydroxyvitamin D_3_ and 50 *μ*M ascorbic acid or with macrophage-colony-stimulating factor (25 ng/ml) and RANKL (50 ng/ml) for 14 days for spleen cells and 21 days for BMCs.

### Fibroblast colony-forming unit assay

The formation of fibroblast colonies positive for alkaline phosphatase (colony-formation unit (CFU)^−^ALP^+^) was assayed with BMCs derived from femurs and tibiae of 10-week-old WT and ColI-Synd2 mice as described.^55^ Briefly, 100 mg/ml ascorbic acid was added to the culture medium on day 4 of culture. After 11 days, cell colonies were stained for ALP activity by using bromochloroindoyl-phosphate/nitroblue tetrazolium chloride (Sigma-Aldrich). Colony formation was determined by use of ImageJ (NIH, Bethesda, MD, USA).

### Apoptosis assay

To test whether osteoblasts could affect the apoptosis of BMCs, osteoblasts from WT or ColI-Synd2 bones were plated in porous inserts (1 *μ*m) and cultured to confluence. BMCs from WT mice were cultured on sterile coverslips in 24-well plates with or without osteoblasts in DMEM/FCS/P/S containing 15% L (control) or Wnt3a-CM for 72 h. The inserts were then removed and 0.5 *μ*M SYTOX Orange nucleic acid stain (Life Technologies, Thermofisher, Waltham, MA, USA) was added to the medium. BMCs exposed to UV light were a positive control for apoptosis induction. The cells were washed and fixed with 4% paraformaldehyde in phosphate-buffered saline and nuclei were stained with 4′,6-diamidino-2-phenylindole (DAPI; Invitrogen). The ratio of Sytox- to DAPI-positive cells was measured under a fluorescent microscope with the Apotome 1 system (Zeiss) and Axiovision software.

### RT-PCR analyses

Total RNA from bone or marrow extracts of 4-month-old mice was reverse-transcribed. Gene expression was analyzed by quantitative real-time PCR (RT-qPCR) with SYBR Green, Light-Cycler 480 (Roche Molecular Diagnostics, Pleasanton, CA, USA), and the following forward and reverse primers:

*HPRT1:* 5‘-TGAAGCGCTGCCAGTATGTTA-3′ 5′-GGTCGCTCAGAGCCTTGTAGA-3′

*Actin:* 5‘-GGCTGTATTCCCCTCCATCG-3′ 5′-CCAGTTGGTAACAATGCCATGT-3′

*hSyndecan-2:* 5′-TTGGCTTTCTCTTTGCAATTTT-3′ 5′-CCTTCATCCTTCTTTCTCTCATGC-3′

*β-catenin*: 5′-GCAGCAGCAGTTTGTGGA-3′ 5′-TGTGGAGAGCTCCAGTACACC-3′

*Wnt3a:* 5′-CTTAGTGCTCTGCAGCCTGA-3′ 5′-ACTGCTCAGAGAGGAGTACTGG-3′

*Wnt11:* 5′-CAGGATCCCAAGCCAATAAA-3′ 5′-TCCAGGGAGGCACGTAGA-3′

*Axin2:* 5′-GCCATTGGCCTTCACACT-3′ 5′-CCATGACGGACAGTAGCGACT-3′

*Wisp1:* 5′-GTGGACATCCAACTACACATCAA-3′ 5′-AACTTCGTGGCCTCCTCTG-3′

*RSPO-2:* 5′-CTGTGAGGTTTCCCTGTGGT-3′ 5′-ACACTCGGCCTCTTCTTCAA-3′

*SOST:* 5′-GGAATGATGCCACAGAGGTCA-3′ 5′-CCCGGTTCATGGTCTGGTT-3′

*DKK1:* 5′-CCGGGAACTACTGCAAAAAT-3′ 5′-CCAAGGTTTTCAATGATGCTT-3′

*SFRP-1:* 5′-GCCACAACGTGGGCTACAA; -3′ 5′-ACCTCTGCCATGGTCTCGTG-3′

*RANKL:* 5′- GAGTGACTTTATGGGAACCCGAT; 5′-GGCCACAGCGCTTCTCAG-3′

*OPG:* 5′-CCATCTGGACATTTTTTGCAAA-3′ 5′-AGTCGGTGAAGCAGGAGTG-3′

*RUNX2:* 5′-TTGACCTTTGTCCCAATGC-3′ 5′-AGGTTGGAGGCACACATAGG-3′

*Bcl2:* 5′-GTACCTGAACCGGCATCTG-3′ 5′-GGGGCCATATAGTTCCACAA-3′

*Bax:* 5′-GTGAGCGGCTGCTTGTCT-3′ 5′-GGTCCCGAAGTAGGAGAGGA-3′

Results are presented as mean *ΔΔ*CT. When the gene expression in WT did not change according the sex, the groups included both male and female RNA extracts. When a sex-related variation was observed only results from males were shown. *HPRT* and/or *Actin* was the reference genes. Results from PCR analyses were excluded when the HPRT level was very different from the other values or because of the poor quality of RNA extracts.

### Statistical analysis

All data are expressed as mean±S.E.M. The significance level was set at *P*<0.05. Two-tailed Student's *t*-test for unpaired samples was used for most statistical analyses except for the measures of syndecan-2 and heparan sulfate overexpression, for which only the increase was tested by one-tailed Student's *t*-test. The *n* values in the legends indicate numbers of independent biological samples used for the analyses.
