# Correction: Osteoblastic heparan sulfate glycosaminoglycans control bone remodeling by regulating Wnt signaling and the crosstalk between bone surface and marrow cells

**DOI:** 10.1038/s41419-018-0830-4

**Published:** 2018-07-16

**Authors:** Rafik Mansouri, Yohann Jouan, Eric Hay, Claudine Blin-Wakkach, Monique Frain, Agnès Ostertag, Carole Le Henaff, Caroline Marty, Valérie Geoffroy, Pierre J Marie, Martine Cohen-Solal, Dominique Modrowski

**Affiliations:** 1 Inserm UMR-1132, BIOSCAR, Paris, France; 2 Université Paris Diderot, Sorbonne Paris Cité, Paris, France; 3 CNRS, UMR 7370, LP2M, Faculté de médecine, 28 avenue de Valombrose, Nice, France; 4 Université Nice Sophia Antipolis, Parc Valrose, Nice, France; 5 CNRS, USR3695, Gif-sur-Yvette, France

Correction to: *Cell Death & Disease*; 10.1038/cddis.2017.287; published online 29 June 2017.

The PDF and HTML versions of the article have been updated to include the Creative Commons Attribution 4.0 International License information.

