# Supplementary figures and images for: Osteoblastic heparan sulfate glycosaminoglycans control bone remodeling by regulating Wnt signaling and the crosstalk between bone surface and marrow cells
Source: Cell Death Dis. 2017 Jun 29;8(6):e2902–. doi: 10.1038/cddis.2017.287 (PMC5520938; doi:10.1038/cddis.2017.287)

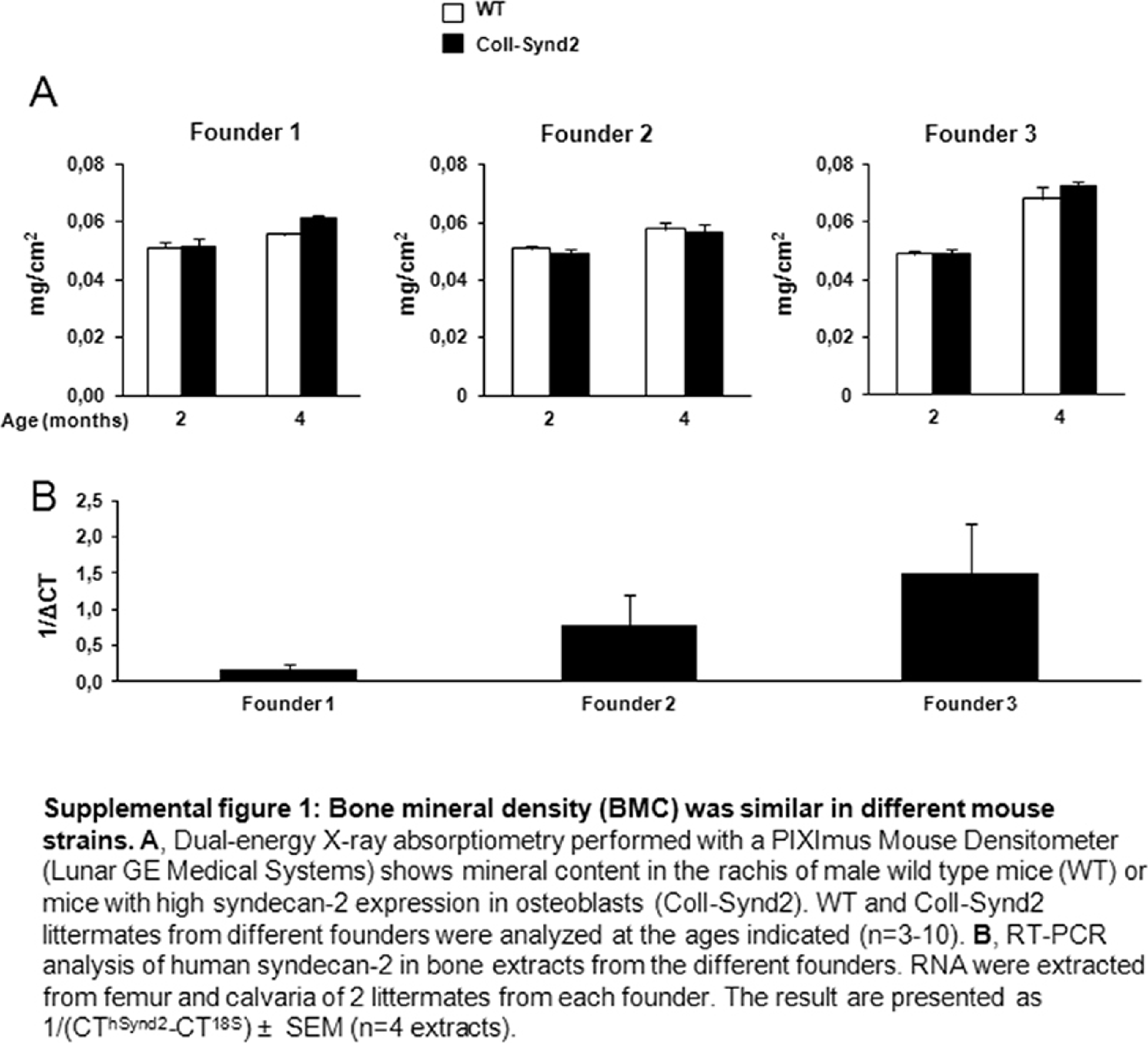

Supplement: Supplementary Figure 1 [file cddis2017287x1.tif]

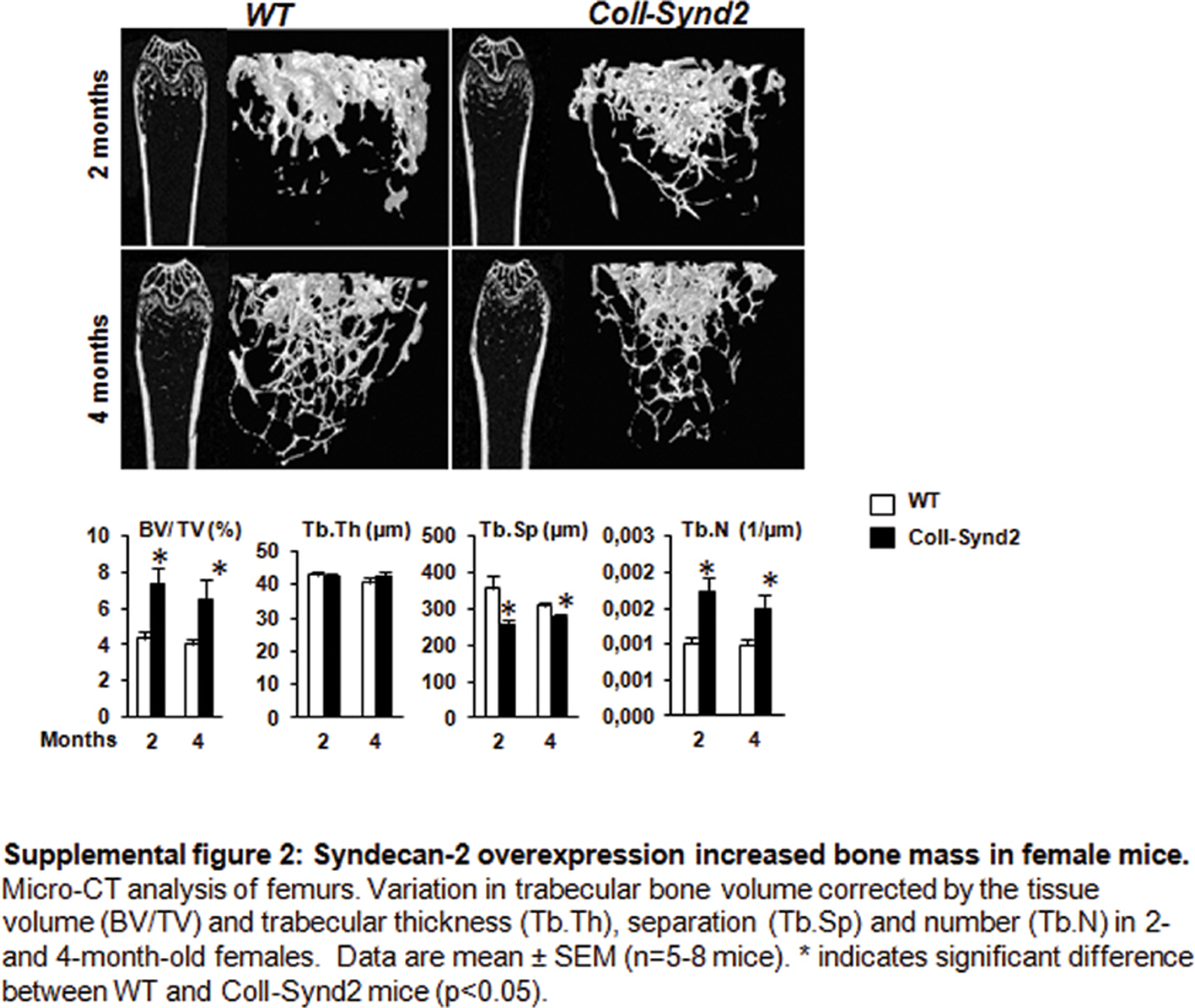

Supplement: Supplementary Figure 2 [file cddis2017287x2.tif]

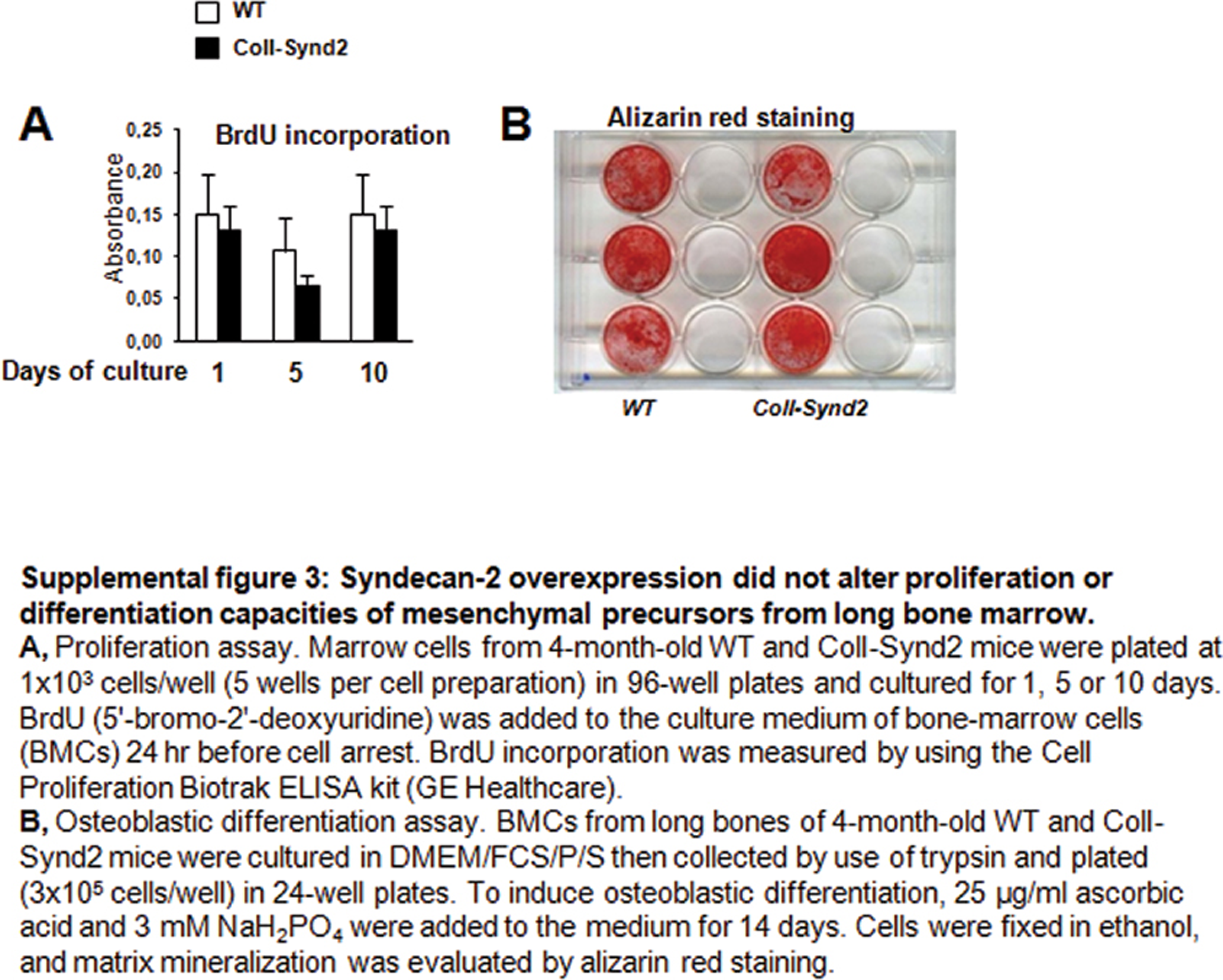

Supplement: Supplementary Figure 3 [file cddis2017287x3.tif]

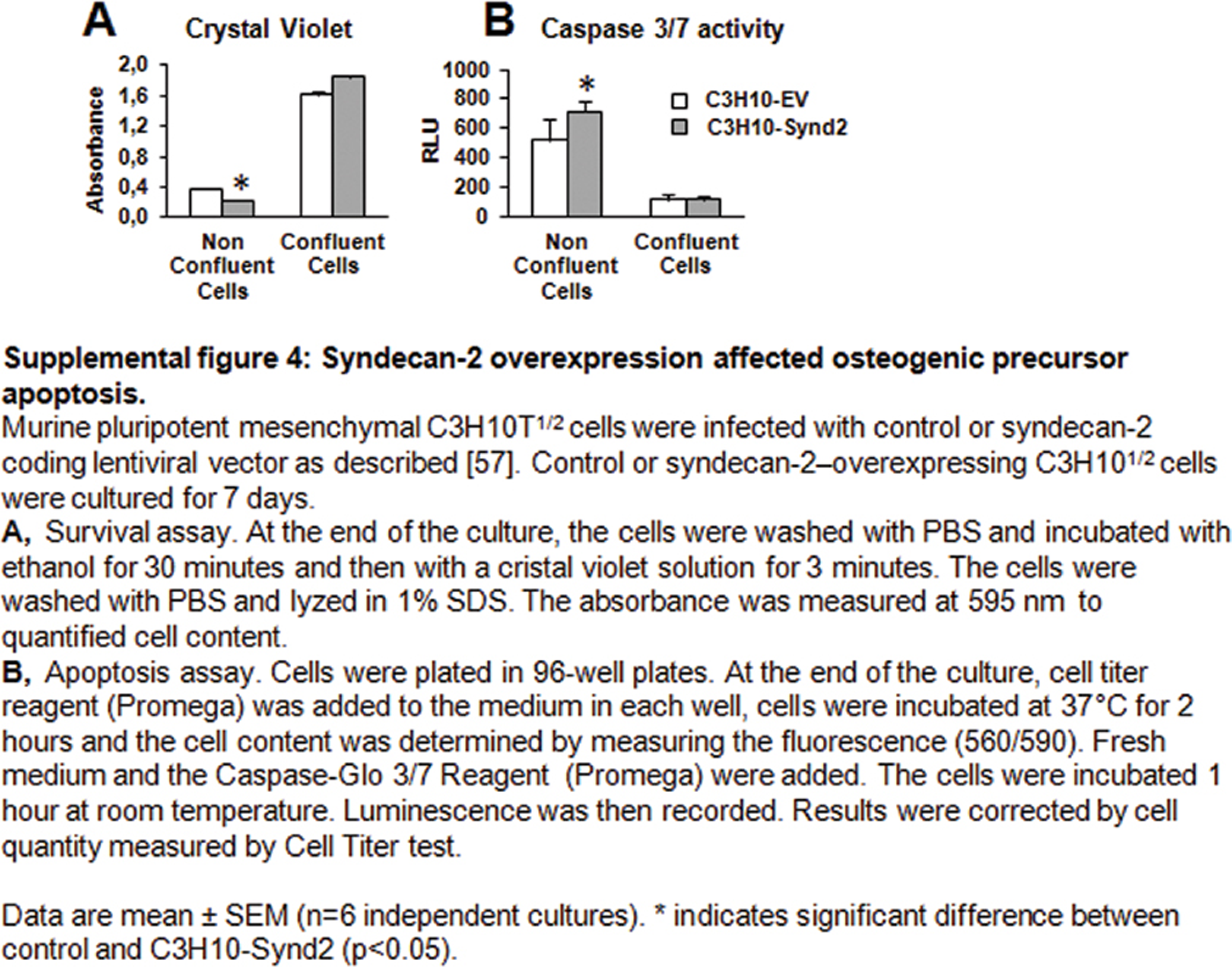

Supplement: Supplementary Figure 4 [file cddis2017287x4.tif]

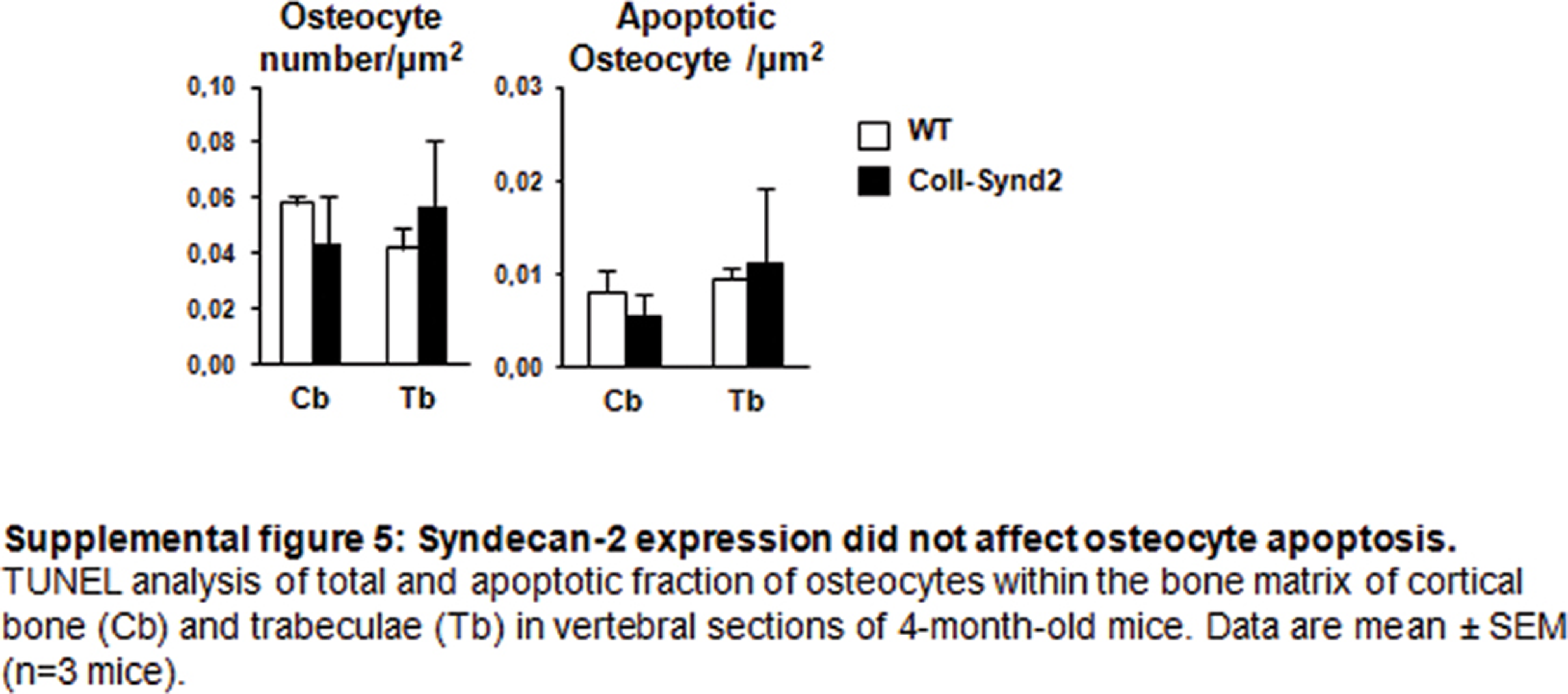

Supplement: Supplementary Figure 5 [file cddis2017287x5.tif]

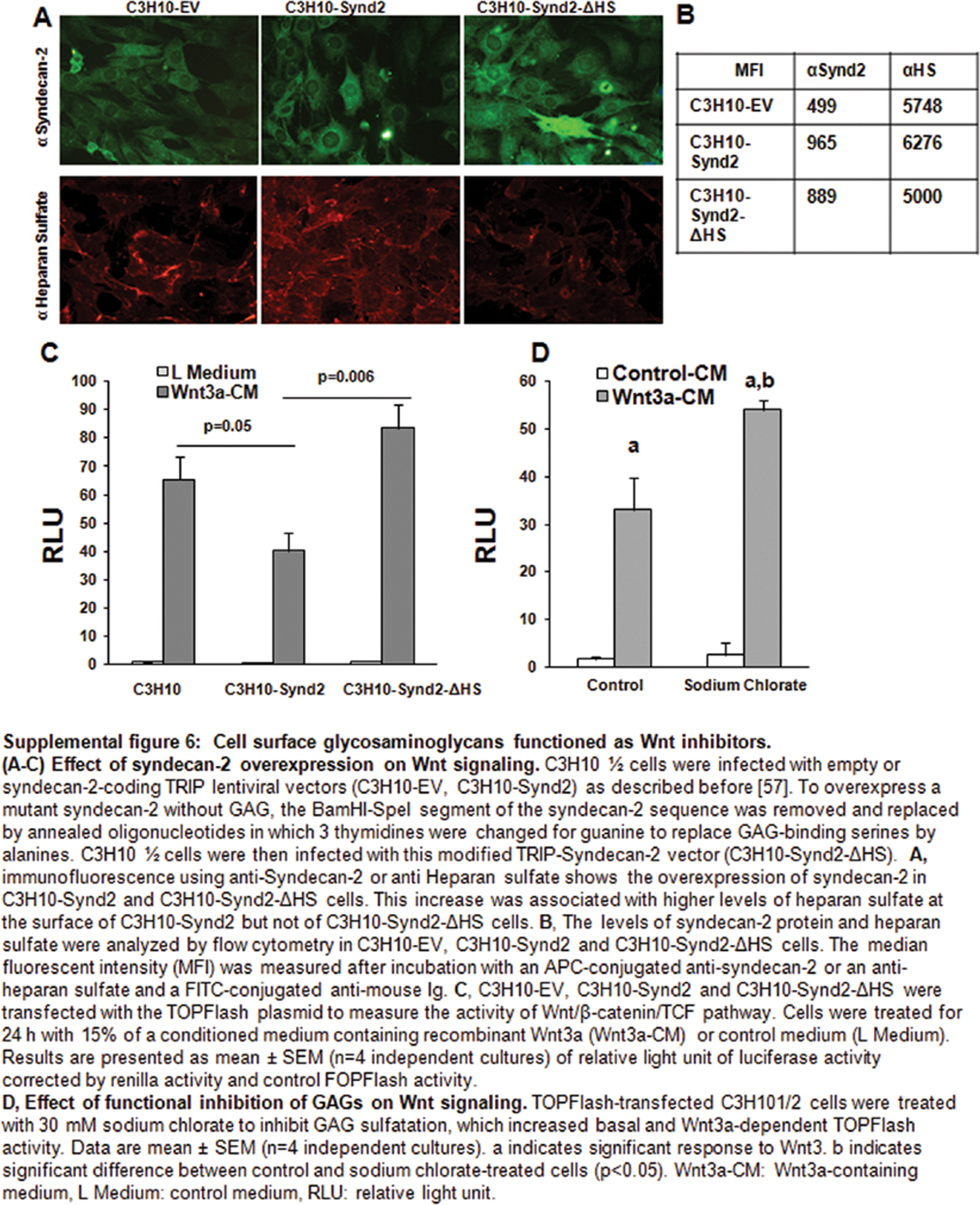

Supplement: Supplementary Figure 6 [file cddis2017287x6.tif]

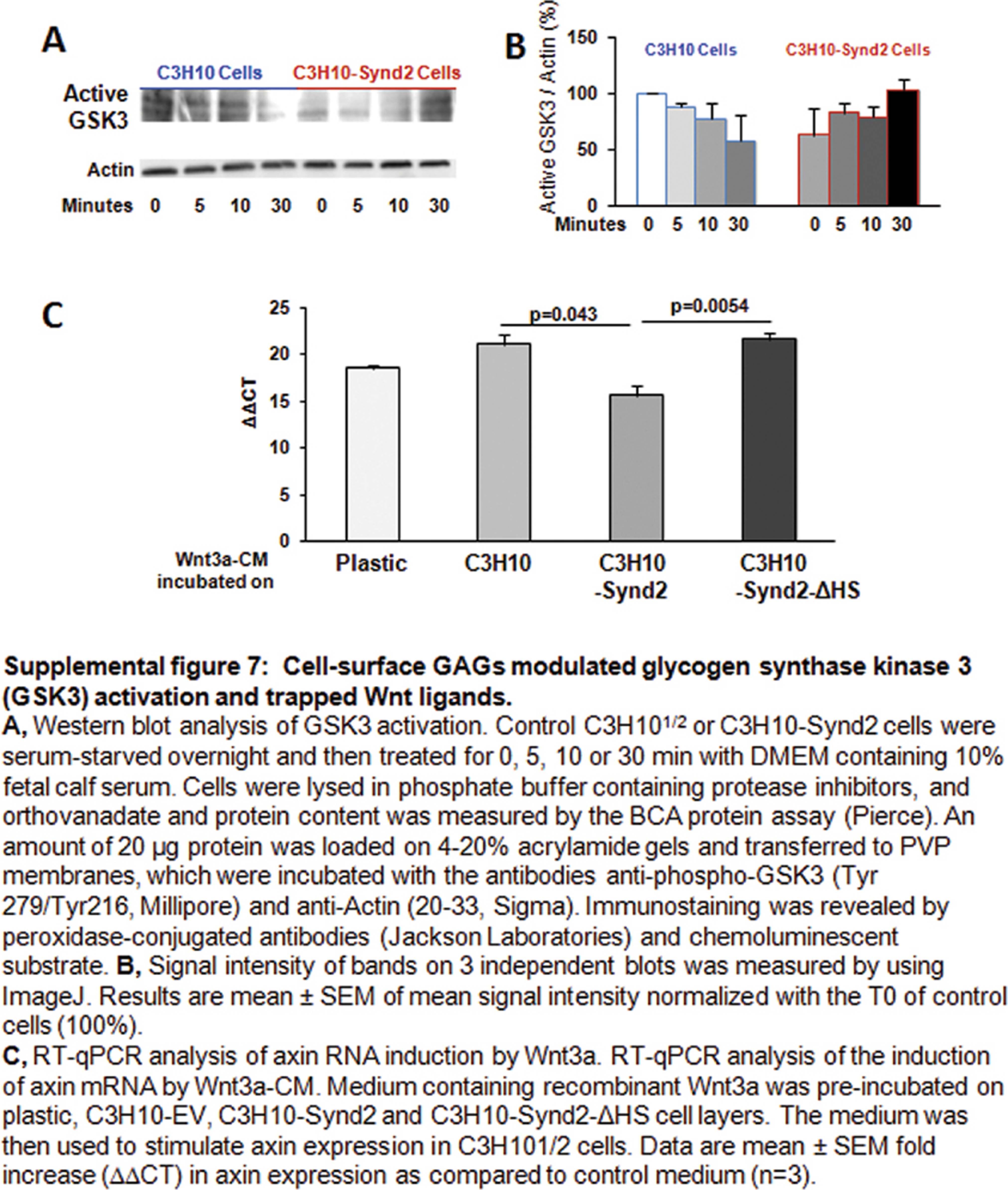

Supplement: Supplementary Figure 7 [file cddis2017287x7.tif]
